# A 4D Proteome Investigation of the Potential Mechanisms of SA in Triggering Resistance in Kiwifruit to *Pseudomonas syringae* pv. *actinidiae*

**DOI:** 10.3390/ijms242417448

**Published:** 2023-12-13

**Authors:** Dong Qu, Fei Yan, Yu Zhang, Lili Huang

**Affiliations:** 1 College of Plant Protection, Northwest A&F University, Xianyang 712100, China; qudong@snut.edu.cn; 2 Shaanxi Provincial Bioresource Key Laboratory, College of Biological Science and Engineering, Shaanxi University of Technology, Hanzhong 723000, China; yanfei@snut.edu.cn (F.Y.); yuzhang20160315@outlook.com (Y.Z.); 3 Qinba State Key Laboratory of Biological Resources and Ecological Environment, Hanzhong 723001, China

**Keywords:** kiwifruit plant, salicylic acid, bacterial canker, proteome

## Abstract

Kiwifruit bacterial cankers caused by *Pseudomonas syringae* pv. *actinidiae* (Psa) are a serious threat to the kiwifruit industry. Salicylic acid (SA) regulates plant defense responses and was previously found to enhance kiwifruit’s resistance to Psa. However, the underlying mechanisms of this process remain unclear. In this study, we used 4D proteomics to investigate how SA enhances kiwifruit’s resistance to Psa and found that both SA treatment and Psa infection induced dramatic changes in the proteomic pattern of kiwifruit. Psa infection triggered the activation of numerous resistance events, including the MAPK cascade, phenylpropanoid biosynthesis, and hormone signaling transduction. In most cases, the differential expression of a number of genes involved in the SA signaling pathway played a significant role in kiwifruit’s responses to Psa. Moreover, SA treatment upregulated numerous resistance-related proteins, which functioned in defense responses to Psa, including phenylpropanoid biosynthesis, the MAPK cascade, and the upregulation of pathogenesis-related proteins. We also found that SA treatment could facilitate timely defense responses to Psa infection and enhance the activation of defense responses that were downregulated in kiwifruit during infection with Psa. Thus, our research deciphered the potential mechanisms of SA in promoting Psa resistance in kiwifruit and can provide a basis for the use of SA to enhance kiwifruit resistance and effectively control the occurrence of kiwifruit bacterial cankers.

## 1. Introduction

Kiwifruit, 90% of which is produced in China, New Zealand, and Italy, has been popularized due to its high nutritional value. Rich in protein, fatty acids, vitamin C, and a variety of mineral elements, as well as flavonoids, triterpenes, anthraquinones and alkaloids, and other bioactive ingredients [1], kiwifruit also exerts pharmacological effects, such as anti-tumor, anti-viral, and anti-oxidant properties; reduces blood sugar and blood fat; and improves immunity. It thus offers a range of opportunities for development [1]. However, kiwifruit bacterial canker (KBC), a devastating disease caused by *Pseudomonas syringae* pv. *actinidiae* (Psa), is a serious threat to the production and development of kiwifruit. Characterized by its wide range of hosts and strong pathogenicity, Psa causes significant problems for disease management, and KBC has seriously restricted the development of the kiwifruit industry. KBC was first detected in Japan in 1984, resulting in a loss of kiwifruit yield [2,3], and was subsequently isolated from many countries [4,5,6,7]. In China, KBC was first found in Hunan Province in 1986 [8] and rapidly spread to Sichuan, Anhui, and Fujian Provinces [9,10,11]. Kiwifruit bacterial cankers are not always visible, and the majority of symptoms mainly occur in the tree, leading to a high fatality rate among kiwifruit trees. Thus, farmers often miss the optimal treatment period, and the lack of timely treatment has a drastic impact on the kiwifruit yield. Currently, chemical agents and proper field management strategies are used to control the spread of the disease [3]. However, problems represented by fewer types of fungicides, unsatisfactory control effects, environmental pollution, etc., have also limited the effective management of KBC in the field. Moreover, the long-term usage of fungicides will also increase bacterial resistance [12]. Therefore, novel control methods must be developed to promote the sustainable development of the kiwifruit industry.

During their interactions with pathogenic microbes, plants have gradually formed a systematic resistance mechanism to defend against them [13]. Plants can deploy a physical structure composed of a wax layer, lignin, phytoalexins, and other chemical components to defend against pathogens [14]. Typically, plants can regulate the activation of relevant resistance responses to resist pathogen infection by transducing specific hormone signals [15]. When infected by pathogens, plants usually mount a resistance response through the positive and negative regulation of the abscisic acid (ABA), salicylic acid (SA), jasmonic acid (JA), and ethylene (ETH) signaling pathways [16,17,18]. ABA and SA have been found to be involved in *Arabidopsis* resistance to *Pseudomonas syringae* [17,18]. SA is an important endogenous signaling molecule in plant systems’ acquired resistance signaling pathway [19]. The main pathway of SA synthesis is catalyzed by isobranched acid synthase and pyruvate formic acid lyase, which synthesize approximately 90% of the total SA [20]. Typically, SA synthesis in plants is species-dependent. Phenylalanine ammonia-lyase (PAL) was also found to be involved in SA synthesis and phenylpropanoid biosynthesis in plants [21]. Previous research showed that the overexpression of Ac-miR160d in kiwifruit increased the expression of PAL and the content of SA, together with related phenylpropanoids [22], suggesting that PAL was responsible for SA synthesis in kiwifruit. Salicylic acid performed an important role in transducing the defense signal, eventually activating the expression of pathogenesis-related proteins (PRs) [23,24,25,26]. SA-mediated resistance has been well documented in several plants, including *Arabidopsis thaliana* and tobacco [14]. SA synthesis and accumulation in these plants are rapidly triggered by pathogen attacks [25,26]. Increased endogenous SA accumulation can induce the expression of resistance proteins, as well as resistance genes that function in the SAR response [27,28,29,30]. The accumulation of salicylic acid in plants can enhance their resistance to a broad spectrum of pathogenic bacteria [31]. Meanwhile, blocking the synthesis or accumulation of SA in plants resulted in a loss of resistance [32]. Additionally, treatments with exogenous SA or its analogues (2,6-dichorisonicotinic acid and BTH) have been found to significantly improve plant disease resistance by inducing an SAR response and upregulating PR proteins [33,34,35]. SA treatment can enhance the kiwifruit’s resistance to Psa infection [36]; however, the underlying regulation mechanisms remain unclear.

In this study, we aimed to deploy in-depth 4D proteomics to investigate the effects of SA in enhancing kiwifruit’s resistance to Psa. We found that SA treatment and Psa infection altered the proteomic patterns of kiwifruit and could activate numerous defense responses. Meanwhile, SA caused the timely defense responses of kiwifruit to Psa infection and could attenuate the inhibitory effects on the resistance responses of plants caused by Psa infection, including the MAPK cascade and phenylpropanoid biosynthesis. Moreover, SA could activate numerous pathogenesis-related proteins and pectinase to help kiwifruit to defend itself against Psa infection. Overall, our research deciphered the detailed proteomic responses of kiwifruit caused by Psa infection and SA treatment and improves our understanding of the mechanisms of SA in mediating the resistance of kiwifruit to Psa.

## 2. Results

### 2.1. Foliar Symptoms and Colonization Dynamics in Psa Treatment and SA + Psa Treatment

To observe the phytopathology in Psa treatment and SA + Psa treatment, we performed vacuum infiltration inoculation on leaf discs. Two days later, disease spots appeared on both discs. The disease spots in SA + Psa leaf discs were slow to expand, although more rapid expansion occurred in response to Psa treatment. However, there was no significant difference in the degree of disease between treatments after osmotic inoculation. Then, the colonization of pathogens in Psa and SA + Psa treatment was determined. The number of pathogens in Psa-inoculated leaves reached 2.86 ± 0.12 × 10^7^ CFU g^−1^. The number of pathogens in SA + Psa treatment leaves was only 1.37 ± 0.15 × 10^7^ CFU g^−1^. Therefore, the bacterial density following SA + Psa treatment was only 47.9% of that observed for Psa treatment leaves.

### 2.2. Proteome Analysis of Kiwifruit Following SA and Psa Treatment

**Figure 1:**
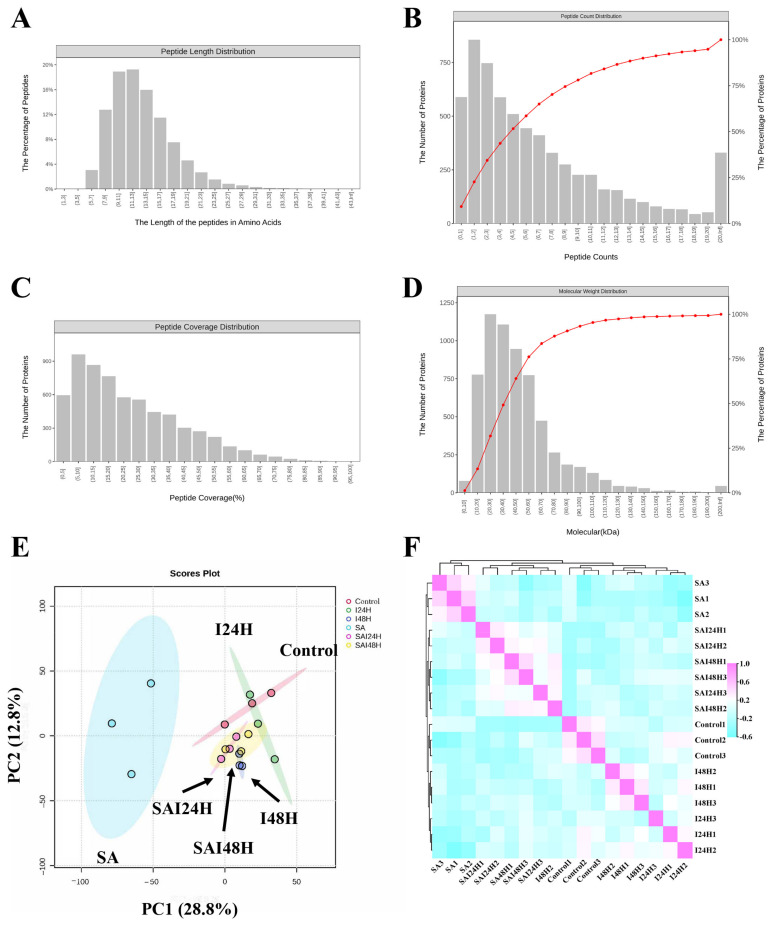
Landscape of proteomic expression in kiwifruit following SA treatment and *Pseudomonas syringae* pv. *actinidiae* (Psa) infection. (**A**) Proteomic quality control and DEPS identification of kiwifruit. Peptide length distribution: most of the peptides were distributed in 7–20 amino acids. (**B**) Peptide number distribution: most proteins corresponded to more than two peptides. (**C**) Protein coverage distribution: the coverage of most proteins was below 30%. (**D**) Protein molecular weight distribution: the molecular weight of the identified protein was present at different stages and was evenly distributed. (**E**) Principal component analyses (PCA) based on proteomic profiles of kiwifruit from different experimental treatments. The ellipse represents the 95% confidence interval. The biological replicates from different treatments are shown in different colors. PCA score plot was constructed based on principal components 1 and 2. (**F**) Unsupervised correlation analysis of samples, showing high similarity between biological replicates.

To investigate the underlying mechanisms of SA in triggering defense responses to *Pseudomonas syringae* pv. *actinidiae*, we collected four groups of samples, including normal growth kiwifruit (control group), SA-treated kiwifruit (SA group), Psa-inoculated kiwifruit (24 h and 48 h post inoculation, I24H and I48H), and kiwi fruit inoculated with Psa after fourteen days of SA pretreatment (24 h and 48 h post inoculation, SAI24H and SAI48H), for 4D proteome analysis. We obtained 6374 proteins from 18 samples from the 4D proteomics sequencing data from all treatments (Table S1), and based on the results of the database search, a quality control analysis of peptides was carried out on kiwifruit samples from all treatments. The results showed the distribution of peptide lengths in 7–20 amino acids and the quality control requirements were met, being consistent with the general rules based on enzymatic digestion and mass spectrometry fragmentation (Figure 1A). Additionally, one protein corresponded to multiple specific peptides, supporting the accuracy and credibility of the quantification results (Figure 1B). The coverage of most proteins was below 40%, while the molecular weight of identified proteins was evenly distributed (Figure 1C,D). Thus, the quality control of the proteome sequencing met the requirements. To obtain an overview of the changes in the proteomic pattern of kiwifruit following SA treatment and Psa infection, we performed PCA analysis on all proteomic profiles of kiwifruit (Figure 1E). The PCA plots constructed using the top two components (principal components 1 and 2) showed significant variations in proteomic patterns among all groups, while PC1 and PC2 explained 28.8% and 12.8% of the variation associated with our treatments (Figure 1E). Typically, SA-treated samples showed observable separation from the control group (control), suggesting that SA induced significant changes in the proteome expression patterns of kiwifruit (Figure 1E). Additionally, samples of kiwifruit following Psa infection at 24 hpi (I24H) overlapped with control samples, whereas I48 samples exhibited distinct differences from control samples, suggesting that Psa infection mainly induced changes in the proteomic pattern of kiwifruit at 48 h post-inoculation (Figure 1E). Additionally, when comparing I24H, SAI24H and the control, we identified distinct changes in the proteome patterns of kiwifruit in SAI24H vs. control, suggesting that SA might promote the activation of defense events in kiwifruit in response to Psa infection (Figure 1E). Additionally, the unsupervised hierarchical clustering of all samples showed a consensus with the PCA result, showing that observable changes in the proteomic pattern of kiwifruit were induced by the SA treatment and Psa infection (Figure 1E,F). Meanwhile, both algorithms showed excellent repeatability between biological replicates, confirming the credibility of our proteomic profiles (Figure 1E,F). Together, these results suggest that SA treatment and Psa infection will alter the proteomic patterns of kiwifruit, potentially leading to changes in the resistance and the disease degree of kiwifruit.

### 2.3. Pseudomonas syringae pv. actinidiae (Psa) Infection Induced Dramatic Variations in Proteome of Kiwifruit

**Figure 2:**
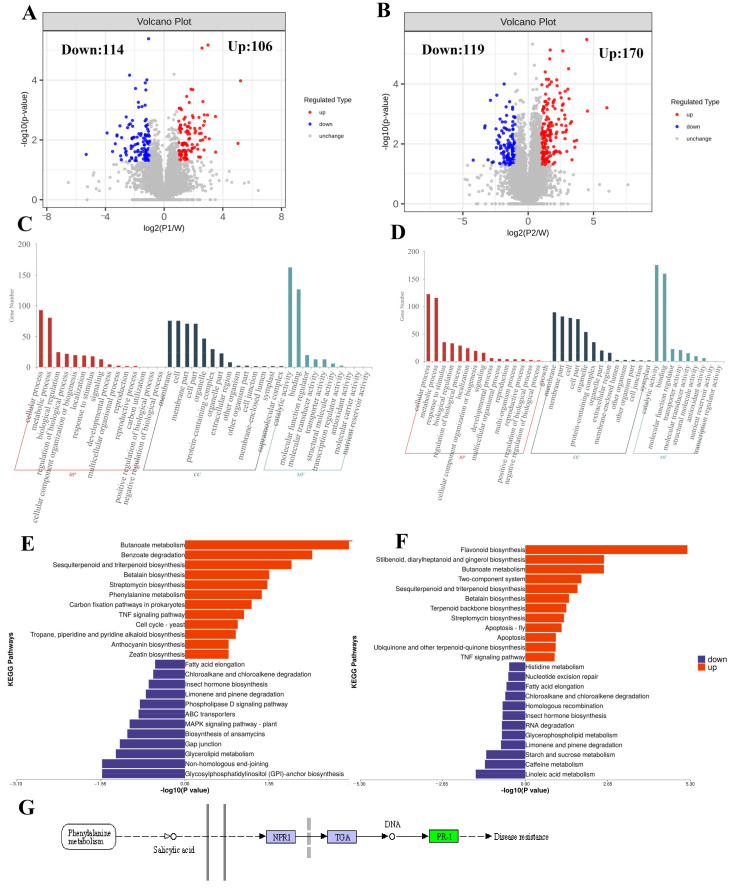
Proteomic variations in kiwifruit following *Pseudomonas syringae* pv. *actinidiae* (Psa) challenge. (**A**,**B**) Volcano plot shows the differentially expressed proteins (DEPs) with fold change ≥ 1 and *p* ≤ 0.05 in kiwifruit following Psa infection at 24 (**A**) and 48 hpi (**B**). The horizontal coordinate represents the multiple changes (log2 fold change), and the vertical coordinate represents the *p*-value of Student’s *t*-test (−log 10 *p*-value). The up- and downregulated proteins are shown in red and blue, respectively. (**C**,**D**) Gene ontology enrichment analysis of DEPs from I24H vs. control (**C**) and I48H vs. control (**D**) pairwise comparisons. GO terms are categorized according to molecular function, biological process and cellular component. (**E**,**F**) The pathway enrichment analysis on DEPs from I24H vs. control (**E**) and I48H vs. control (**F**) pairwise comparisons. The enriched pathways for upregulated and downregulated DEPs are shown in red and purple, respectively. (**G**) SA signaling pathway is involved in the responses of kiwifruit to Psa infection.

To identify the detailed changes in kiwifruit following Psa challenge, we compared the proteomic profiles of kiwifruit at 24 (I24H) and 48 (I48H) hours post-Psa inoculation. We identified 220 differentially expressed proteins (DEPs) using a threshold of *p* < 0.05 and fold change > 2.0 in the I24H vs. control comparison; 106 upregulated proteins and 114 downregulated proteins were also identified (Figure 2A). Subcellular location analysis showed that these DEPs mainly performed their functions in the cytoplasmic, nuclear and plasma membrane (Table S2). Then, GO enrichment analysis was performed on these 220 DEPs, and 99 GO terms were significantly enriched against them (Figure 2C). The cellular component results showed that these DEPs were mainly involved in the chromosome, external encapsulating structure and cell wall (Figure 2C). Among 36 significant terms relevant to molecular functions, the top 10 terms were enzyme inhibitor activity, isoprenoid binding, alcohol binding, abscisic acid binding, hormone binding, phosphatase inhibitor activity, protein phosphatase inhibitor activity, signaling receptor activity, lipid binding and phenylalanine ammonia-lyase activity (Figure 2C). In terms of biological progress, 60 related terms were significantly overrepresented against these DEPs, including the cellular response to lipids, hormone-mediated signaling pathway, defense response, signal transduction, cell wall biogenesis, xyloglucan metabolic process, secondary metabolic process, hemicellulose metabolic process, phenylpropanoid metabolic process and cell wall organization (Figure 2C). The DEPs associated with Psa infection at 24 hpi were mainly involved in the phenylpropanoid pathway and cell wall organization (Figure 2C) and these were the main responses of plants when defending against pathogen attacks [37]. Further pathway analysis showed that 106 upregulated proteins in I24H vs. control were mainly involved in butanoate metabolism, benzoate degradation, flavonoid biosynthesis, sesquiterpenoid and triterpenoid biosynthesis, phenylpropanoid biosynthesis, terpenoid backbone biosynthesis, spliceosome, valine, leucine and isoleucine degradation, lysine degradation and betalain biosynthesis (Figure 2E; Table S1). Numerous proteins involved in the resistance pathways of plants were activated by Psa infection, especially flavonoid biosynthesis, sesquiterpenoid and triterpenoid biosynthesis and phenylpropanoid biosynthesis (Figure 2E; Table S1). It has been found that phenylpropanoids, flavonoids and terpenoids involved in plant defense respond to pathogens through their antifungal activity [37]. In parallel, a pathway analysis of 114 downregulated proteins showed the significant enrichment of lysine degradation, glycosylphosphatidylinositol (GPI) anchor biosynthesis, glycerolipid metabolism, tryptophan metabolism, the MAPK signaling pathway, ABC transporters and the phospholipase D signaling pathway (Figure 2E; Table S1). Notably, we also observed the activation of the SA signaling pathway in kiwifruit in response to Psa infection (Figure 2G), suggesting the importance of the SA-mediated defense response in kiwifruit’s response to Psa.

Subsequently, we analyzed the changes in proteome function in the I48H vs. control comparison, and 289 DEPs associated with Psa infection were identified, with 119 down- and 170 upregulated DEPs (Figure 2B). These 289 DEPs in the I48H vs. control comparison mainly functioned in the cytoplasmic, nuclear, plasma membrane, chloroplast, extracellular, mitochondrial and lysosomal domains of the kiwifruit cell (Table S1). These DEPs were collected for KEGG pathway and gene ontology (GO) enrichment analyses (Table S1; Figure 2D,F). For GO enrichment analysis, the most significant terms relevant to biological processes, molecular functions and cellular components are shown in Figure 2D (FDR < 0.05). For cellular component terms, the most significant terms were the extracellular region, external encapsulating structure, cell wall and lipid droplet (Figure 2D). For molecular function, 34 terms were significant based on these DEPs, especially enzyme inhibitor activity, isoprenoid binding, hormone binding, organic acid binding, carboxylic acid binding, signaling receptor activity, phosphatase regulator activity, lipid binding and phenylalanine ammonia-lyase activity (Figure 2D). Of the 92 significant terms relevant to biological processes, the top 10 terms were the hormone-mediated signaling pathway, defense response, response to chemicals, response to stress, signal transduction, response to stimulus, secondary metabolic process, cell wall organization, reactive nitrogen species metabolic process and response to biotic stimulus (Figure 2D). We noted that the enrichment of terms relevant to phenylpropanoids was also significant in the I48H vs. control comparison (Figure 2D), supporting their involvement in the defense responses of kiwifruit to Psa. Similarly, the KEGG pathway analysis of 170 upregulated DEPs showed the significant enrichment of phenylpropanoid biosynthesis, flavonoid biosynthesis, stilbenoid, diarylheptanoid and gingerol biosynthesis, plant hormone signal transduction, pentose and glucuronate interconversion, fatty acid degradation, the spliceosome, phenylalanine metabolism, sesquiterpenoid and triterpenoid biosynthesis and betalain biosynthesis (Figure 2F; Table S1). The pathway analysis results also suggested that phenylpropanoid and flavonoid biosynthesis were involved in the defense responses of kiwifruit to Psa infection at 48 hpi (Figure 2F; Table S1). Of note, plant hormone signal transduction represented by the SA signal was also identified in the I48H vs. control comparison (Figure 2F,G; Table S1), suggesting that SA might regulate resistance in kiwifruit in response to Psa infection. By contrast, downregulated proteins were also involved in the lysosome, linoleic acid metabolism, plant hormone signal transduction, starch and sucrose metabolism, the MAPK signaling pathway and phenylpropanoid biosynthesis, indicating that the abundance of some related proteins was reduced at 48 hpi post-Psa inoculation (Figure 2F; Table S1). Overall, these results suggested that the phenylpropanoid biosynthesis and SA signaling genes were involved in kiwifruit’s response to Psa challenge.

### 2.4. Exogenous SA Treatment Altered the Proteomic Pattern of Kiwifruit

**Figure 3:**
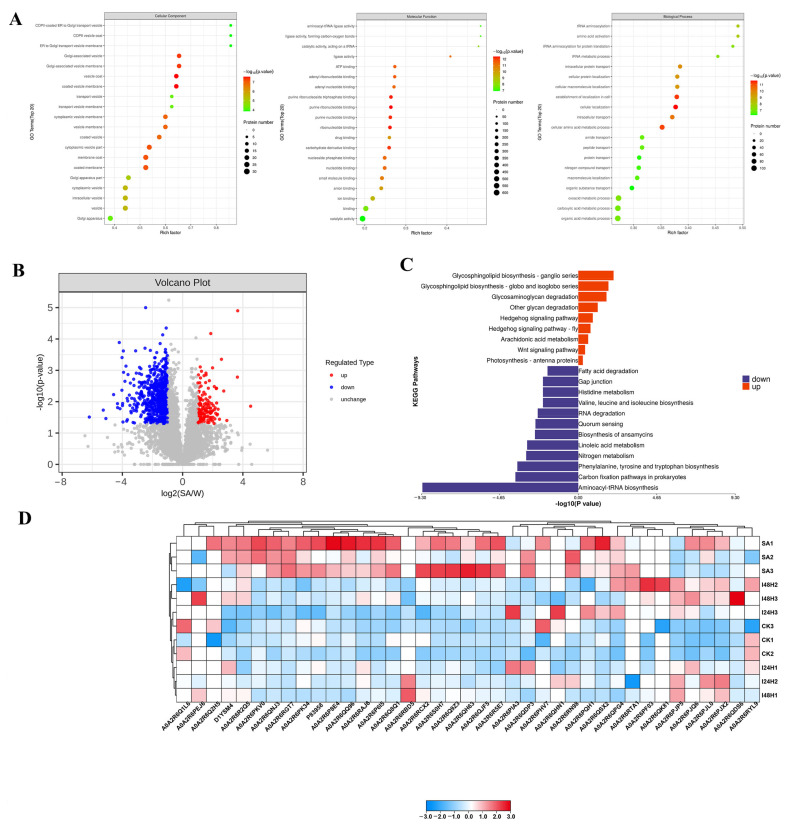
SA treatment alters the proteomic pattern of kiwifruit. (**A**) Scatter plot of the GO enrichment analysis of DEMs associated with SA treatment. The number of proteins in each GO term is represented by the plot size. The plot color indicates the significant differences of each GO term. (**B**) Volcano plot shows the DEPs with log_2_fold change ≥ 2 and *p* ≤ 0.05 in SA vs. control pairwise comparison. Each point in the volcano diagram represents a protein, the horizontal coordinate represents the multiple changes (log_2_ fold change), and the vertical coordinate represents the *p*-value of Student’s *t*-test (−log_10_ *p*-value). The up- and downregulated proteins are shown in red and green, respectively. (**C**) The pathway enrichment results based on DEPs of kiwifruit in SA vs. control comparison. (**D**) Heatmap displays the expression levels of resistance genes in kiwifruit after SA treatment and Psa infection. The high and low levels of proteins in each biological replicate are shown in red and blue, respectively.

To investigate the effects of SA in promoting resistance in kiwifruit to Psa, we compared the proteomic profiles of kiwifruit before and after SA treatment. In total, 833 DEPs were identified in the SA vs. control comparison, and these DEPs were mainly distributed in the cytoplasmic, nuclear, chloroplast, plasma membrane, mitochondrial and extracellular areas in kiwifruit cells (Table S3). The GO enrichment analysis of these DEPs revealed the significant enrichment of 48 terms relevant to cellular components, such as coated vesicle membrane, vesicle coat, Golgi-associated vesicle membrane, Golgi-associated vesicle, coated membrane, membrane coat, cytoplasmic vesicle part, vesicle membrane, cytoplasmic vesicle membrane, coated vesicle, cytoplasmic vesicle and Golgi apparatus part (Figure 3A). The DEPs associated with SA treatment mainly exhibited purine ribonucleotide binding, ribonucleotide binding, carbohydrate derivative binding, ATP binding, ligase activity, ion binding, catalytic activity, ATPase activity, calcium ion transmembrane transporter activity, ubiquitin–protein transferase activity, shikimate 3-dehydrogenase (NADP+) activity, chloroplast protein-transporting ATPase activity, UDP-glycosyltransferase activity and calmodulin binding activity (Figure 3A). Meanwhile, these DEPs were mainly involved in cellular localization, cytoskeleton organization, the chorismate metabolic process, the glutamate metabolic process, the shikimate metabolic process, chloroplast localization, chloroplast organization, organelle organization, carbon fixation and the L-phenylalanine metabolic process (Figure 3A). Among these DEPs, 149 DEPs were upregulated in kiwifruit in response to SA treatment, and 684 DEPs were downregulated by SA (Figure 3B). Further KEGG pathway analysis showed that the upregulated DEPs were mainly involved in sphingolipid metabolism, phenylpropanoid biosynthesis, glycosaminoglycan degradation, the lysosome, galactose metabolism, amino sugar and nucleotide sugar metabolism, taurine and hypotaurine metabolism, the spliceosome and the MAPK signaling pathway (Figure 3C; Table S4). The function of phenylpropanoid biosynthesis and the MAPK signaling pathway in plant resistance to biotic stress has been well documented [37,38]. Thus, SA might strengthen kiwifruit’s resistance to Psa by activating phenylpropanoid biosynthesis and the MAPK signaling pathway (Table S4). Furthermore, we analyzed the expression levels of resistance-related genes in kiwifruit under SA treatment and found that the upregulation of proteins relevant to pathogenesis-related proteins (PRs), secondary metabolism represented by phenylpropanoids, JA-mediated defense responses and receptors was triggered in kiwifruit as a result of SA treatment (Figure 3D). These results suggest that SA could enhance the resistance in kiwifruit, especially through activating the MAPK signaling pathway and phenylpropanoid biosynthesis.

### 2.5. SA Promoted the Activation of Resistance Responses in Kiwifruit in Responses to Psa Infection

**Figure 4:**
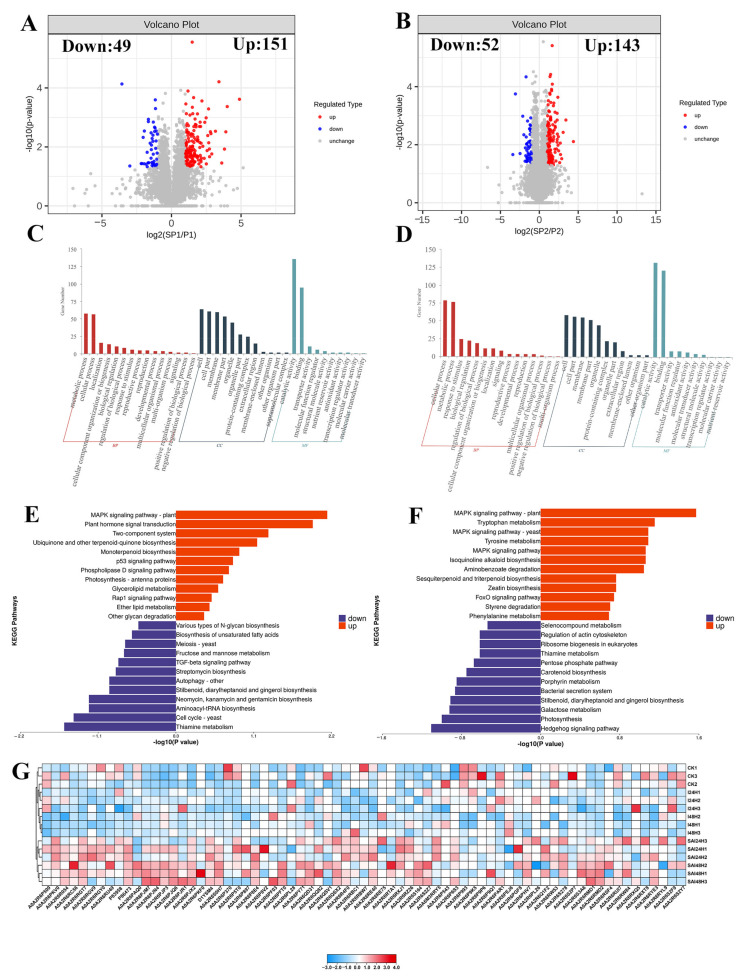
Defense responses of kiwifruit to Psa infection are strengthened by SA treatment. (**A**,**B**) Volcano plot shows the differentially expressed proteins (DEPs) in SAI24H vs. I24H (**A**) and SAI48H vs. I48H (**B**). (**C**,**D**) GO enrichment analysis of DEPs from SAI24H vs. I24H (**C**) and SAI48H vs. I48H (**D**). (**E**,**F**) The pathway enrichment analysis of DEPs from SAI24H vs. I24H (**E**) and SAI48H vs. I48H (**F**). The enriched pathways related to upregulated and downregulated DEPs are shown in red and purple, respectively. (**G**) Activation of resistance-related proteins in kiwifruit via Psa infection is promoted by SA treatment.

Next, we further analyzed the effects of SA on the proteomic pattern of kiwifruit following Psa infection. Based on the threshold of *p* < 0.05 and fold change > 2.0, we identified 200 DEPs in the SAI24H vs. I24H (49 downregulated DEPs and 151 upregulated DEPs) and 195 DEPs in the SAI48H vs. I48H comparisons (52 downregulated DEPs and 143 upregulated DEPs) (Figure 4A,B). The GO enrichment analysis of 200 DEPs in the SAI24H vs. I24H comparison revealed the overrepresentation of 81 GO terms, including the extracellular region, metal ion transmembrane transporter activity, multi-organism reproductive process, regulation of flower development, glucosyltransferase activity, FAD binding, cell wall organization or biogenesis, regulation of shoot system development, UDP-glucose 6-dehydrogenase activity, cell wall organization and ion antiporter activity (Figure 4C). Further KEGG pathway enrichment showed that the 151 upregulated DEPs in SAI24H vs. I24H were mainly involved in the MAPK signaling pathway, plant hormone signal transduction, monoterpenoid biosynthesis, photosynthesis, glycerolipid metabolism, oxidative phosphorylation, phenylpropanoid biosynthesis, lysine degradation and flavonoid biosynthesis (Figure 4E; Table S5). Additionally, the 49 downregulated DEPs mainly contributed to thiamine metabolism, aminoacyl-tRNA biosynthesis, autophagy, starch and sucrose metabolism, amino sugar and nucleotide sugar metabolism, fructose and mannose metabolism and the biosynthesis of unsaturated fatty acids (Figure 4E; Table S5). The MAPK signaling pathway has been found to be involved in plant defense in the response to pathogens, while pathogen challenge could affect the photosynthesis of plants [38,39]. Thus, SA treatment might help plants to activate their pathogen resistance.

Next, we analyzed the DEPs in SAI48H vs. I48H using the GO algorithm and noted the significant enrichment of 148 terms, especially the response to stimulus, reactive oxygen species metabolic process, response to lipids, response to stress, response to hormones, response to organic substances, UDP-galactosyltransferase activity, flower development, phosphatase regulator activity and signal transduction (Figure 4D). Meanwhile, the upregulated proteins in SAI48H vs. I48H were mainly involved in the spliceosome, the MAPK signaling pathway, plant hormone signal transduction, tryptophan metabolism, tyrosine metabolism, zeatin biosynthesis, phenylpropanoid biosynthesis and glycerophospholipid metabolism (Figure 4F; Table S5). In parallel, the downregulated DEPs were primarily involved in photosynthesis, galactose metabolism, carbon fixation in photosynthetic organisms, carotenoid biosynthesis, pyruvate metabolism and the pentose phosphate pathway (Figure 4F; Table S5). Notably, numerous pathways suppressed by Psa infection were activated in kiwifruit following SA treatment, particularly the MAPK signaling pathway and plant hormone signal transduction (Figure 4E,F; Table S5), suggesting that SA could attenuate the effects of Psa in inhibiting plant resistance. Importantly, the expression of resistance genes relevant to hormone signaling, pathogenesis-related proteins, secondary metabolites and receptors was at a low level in Psa-challenged kiwifruit, whereas their expression was triggered to high levels in SA-treated kiwifruit following Psa infection (Figure 4G). This indicates that SA could attenuate the inhibitory effects on plant resistance via Psa infection.

We further analyzed the effects of SA on the plant response following Psa infection. The results showed that SA promoted the upregulation of some defense-related proteins involved in pathogenesis-related proteins, defense signaling and secondary metabolism in kiwifruit following Psa infection. However, these proteins were downregulated in kiwifruit following Psa infection without SA treatment. Thus, we propose that SA could attenuate the inhibitory effects on plant resistance via Psa infection.

### 2.6. SA Promoted Multi-Responses Relevant to Resistance to Protect Kiwifruit from Psa Infection

**Figure 5:**
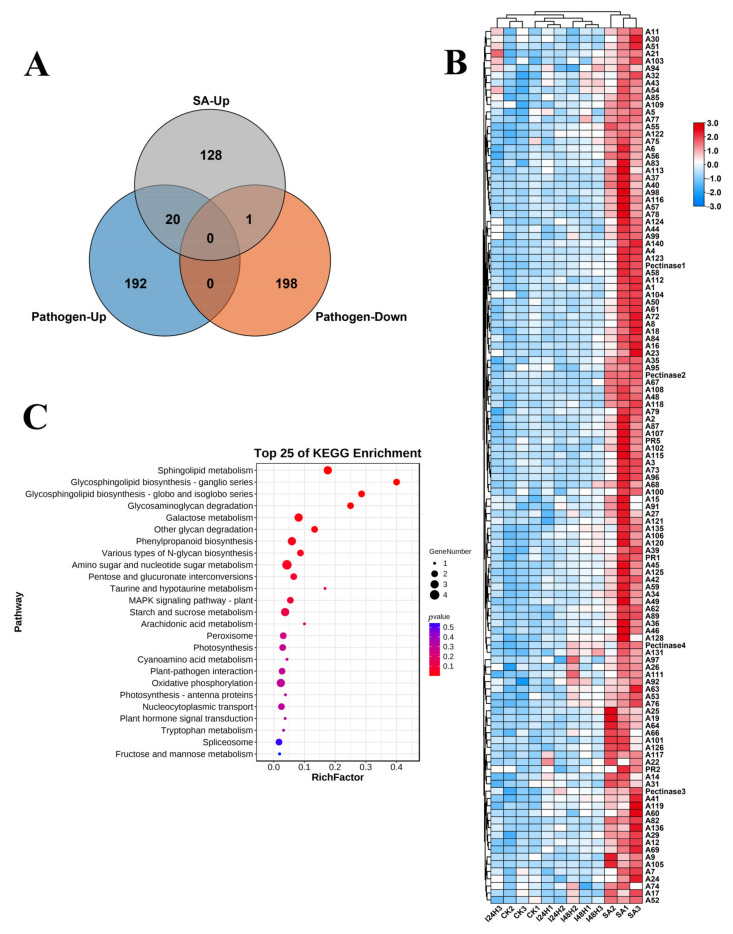
SA treatment promotes the expression of pathogenesis-related proteins and pectinase to enhance kiwifruit resistance to Psa. (**A**) Venn diagram identifying the overlapped DEPs associated with SA treatment and Psa infection. (**B**) Heatmap displaying the relative levels of DEPs in kiwifruit after SA treatment and Psa infection. The high expression levels of proteins in each biological replicate are shown in red. (**C**) Heatmap displaying the expression levels of DEPs activated by both Psa infection and SA treatment. High expression levels of proteins are shown in red.

For a more detailed investigation of the effects of SA in enhancing kiwifruit resistance to Psa infection, we compared the DEPs associated with both SA treatment and Psa infection. Both SA and Psa infection commonly triggered the upregulation of 20 proteins in kiwifruit. The KEEG pathway analysis showed that these 20 DEPs were responsible for sphingolipid metabolism, glycosphingolipid biosynthesis, glycosaminoglycan degradation, galactose metabolism, other glycan degradation, phenylpropanoid biosynthesis, various types of N-glycan biosynthesis, amino sugar and nucleotide sugar metabolism, pentose and glucuronate interconversion, taurine and hypotaurine metabolism and the MAPK signaling pathway (Figure 5C; Table S6). Moreover, most resistance-related genes were activated in SA-treated kiwifruit, supporting SA’s enhancement of Psa resistance (Figure 5B). We also noted that both SA treatment and Psa infection could activate phenylpropanoid biosynthesis and the MAPK signaling pathway in kiwifruit (Figure 5C; Table S6). Thus, phenylpropanoid biosynthesis and the MAPK signaling pathway were the hub pathways in kiwifruit in terms of defending against Psa infection, while SA treatment could activate their activity in vivo, making them highly resistant to Psa infection. We also identified the enrichment of plant–pathogen interaction and plant hormone signal transduction based on these 20 DEPs (Figure 5C; Table S6). Numerous pathogenesis-related proteins are involved in both plant–pathogen interaction and plant hormone signal transduction pathways, including PR2 (glucan endo-1,3-beta-glucosidase), PR5 (thaumatin-like protein) and PR1 (Figure 5B). Importantly, Psa infection triggered a slight upregulation in the expression levels of PR2, PR5 and PR1, and the dramatic upregulation of their levels was also induced by SA in kiwifruit (Figure 5B). Thus, we determined that the upregulated expression of PR2, PR5 and PR1 could be induced by SA, leading to high pathogen resistance in plants. We also observed the significant involvement of cell wall-relevant proteins, particularly pectinase, in SA-treated kiwifruit (Figure 5B); these proteins could strengthen the physical barriers of plant cells to defend against pathogen infection.

## 3. Discussion

The kiwifruit is an important fruit, although its industrial development has been limited by kiwifruit bacterial canker (KBC) caused by *Pseudomonas syringae* pv. *actinidiae* [2,3]. Salicylic acid (SA) is an important hormone involved in regulating plants’ defense responses [40]. Moreover, SA has been reported to enhance plants’ resistance to pathogenic bacteria [40], and understanding the underlying mechanisms is of great significance to the usage of SA in KBC management. In this study, we deciphered the detailed proteomic landscape of kiwifruit following SA treatment and Psa infection using 4D proteomics. We found that both SA and Psa induced significant changes in the proteomic patterns of kiwifruit. Psa infection mainly activated proteins involved in phenylpropanoid biosynthesis and hormone signal transduction, whereas it inhibited the MAPK cascade in kiwifruit at 24 and 48 hpi. In terms of hormone signal transduction, SA-related proteins were highly upregulated by Psa infection, suggesting that SA regulates the defense responses of kiwifruit to Psa. SA mainly upregulated proteins involved in phenylpropanoid biosynthesis and the MAPK cascade. Moreover, we found that SA-treated kiwifruit responded to the pathogen infection earlier (24 hpi), suggesting that SA treatment might enhance kiwifruit’s resistance through inducing timely defense responses. Meanwhile, numerous resistance-related proteins that were downregulated in Psa-infected kiwifruit were upregulated in SA-treated kiwifruit following Psa infection, indicating that SA could attenuate the inhibitory effects of Psa in kiwifruit resistance. Importantly, we also observed that SA upregulated numerous pectinases and pathogenesis-related proteins, including PR2, PR5 and PR1, in kiwifruit, which could function downstream of the SA-mediated defense responses in kiwifruit when faced with Psa infection. Overall, we elucidated the potential mechanisms of SA in enhancing the resistance of kiwifruit via regulating multilayer responses, which provides a theoretical basis for the use of SA to manage kiwifruit bacterial canker.

Kiwifruit bacterial canker caused by *Pseudomonas syringae* pv. *actinidiae* (Psa) is a common problem in kiwifruit [2,3], and it has had devastating effects on the production and cultivation of kiwifruit in recent years [2,3]. The current strategy of using chemical agents for Psa management fails to effectively control the disease occurrence and may eventually lead to numerous environmental problems [3]. With the progression of molecular breeding technology, developing disease-resistant varieties has become an effective means of disease management. However, the limited understanding of the defense mechanisms of kiwifruit against Psa has limited the breeding of resistant kiwifruit materials. During their long-term interaction with pathogenic microbials, plants have evolved complex regulatory networks of defenses to recognize and combat invading microbes and herbivorous insects, including basal resistance and R-gene-mediated resistance [16,17,18,41,42,43,44]. In this study, we performed 4D proteome analysis on kiwifruit to investigate its defense against Psa infection. We found that Psa infection activated numerous basal resistance events, including hormone signaling transduction, the MAPK cascade, the synthesis of phytoalexins, the activation of pathogenesis-related proteins and the burst of reactive oxygen species. Numerous genes that have been proven to be involved in plant resistance to pathogenic microbials were upregulated by SA and Psa treatment, especially a selection of pathogenesis-related proteins represented by PR1, PR5 and PR4 (accessions: P83958, P86473) (Figure 4G). Additionally, our data showed that proteins relevant to the MAPK cascade were also involved in the SA-promoted resistance to Psa in kiwifruit. Mitogen-activated protein kinase (MAPK) cascades are highly conserved signaling modules downstream of receptors/sensors that transduce extracellular stimuli into intracellular responses in eukaryotes. Plant MAPK cascades play pivotal roles in plant defense signaling against pathogen attacks [38]. Numerous studies have revealed that SA can activate the MAPK cascade in plants, thus triggering downstream resistance events [45]. Thus, the activation of the MAPK cascade was also essential to the high resistance of SA-treated kiwifruit to Psa infection. Although the function of the related proteins in these events is in need of further study, our results provide a genetic resource for the breeding of resistant kiwifruit cultivars. Plant defense systems are tightly coordinated through the activities of several phytohormones, particularly salicylic acid (SA). SA is a defense signal molecule that participates in the resistance against biotrophic and hemibiotrophic pathogens by inducing SAR [46,47]. SAR, one of the most commonly induced defense mechanisms, is typically triggered when plants are invaded locally by pathogens, and it provides long-lasting and broad-spectrum protection for the whole plant [48]. The establishment of SAR involves the generation and transport of signals to uninfected distal tissues, which is highly dependent on the higher levels of in vivo SA, since the blocking of SA accumulation inhibits SAR induction [49,50]. When plants are attacked by a pathogen, the SA concentrations increase from the basal level to a high level. It was found that the overexpression of salicylate hydroxylase (NahG) in tobacco and Arabidopsis thaliana could inhibit the accumulation of SA in plants, thereby reducing the resistance of plants to pathogens, supporting the idea that SA is an important plant defense hormone [51,52,53]. Moreover, in many plants, the exogenous application of SA can also induce SAR and enhance plants’ resistance to disease [54]. Here, we found that exogenous SA application could activate numerous resistance events in kiwifruit, including the upregulation of pathogenesis-related proteins and the activation of phenylpropanoid biosynthesis and the MAPK cascade. Phenylpropanoids and phytoalexins could function as a physical barrier to defend against pathogen infection [37,38]. Thus, the activation of phenylpropanoid biosynthesis due to SA treatment could directly enhance kiwifruit’s resistance to Psa infection. Overall, these studies provide a theoretical basis for the use of SA to enhance kiwifruit’s resistance to Psa infection, and they also provide genetic resources for the breeding of resistant cultivars.

## 4. Materials and Methods

### 4.1. Sample Preparation and Collection

The experimental materials, grafted four-year-old resistant-variety *Actinidia deliciosa* var. Xuxiang kiwifruit plants, were derived from the kiwifruit orchard of Shaanxi Fruit Group Mianxian Co., Ltd., Hanzhong City, China (longitude: E 106° 48′00″, latitude: N 33° 09′00″). Sixty Xuxiang kiwifruit plants were divided into four groups.

In the first group (normal growth control group) and the second group (I group), the adaxial and abaxial leaf surfaces were sprayed with water until dripping occurred. The third group (SA treatment, SA group) and the fourth group’s (SAI group) adaxial and abaxial leaf surfaces were sprayed with salicylic acid (2.5 mmol/L) until dripping. This operation was repeated seven days later. Subsequently, the leaves were collected seven days after the second round of spraying with water and SA. Thirty leaves from fifteen plants for each group, all fully unfolded newborn leaves with uniform growth and size, were selected [55]. All leaves from each group were disinfected with 0.6% NaClO solution for 5 min (during which close attention was paid to avoiding damage to the leaves during the experiment) and rinsed with aseptic water. The residual water on the leaf surface was dried with filter paper. Each group of leaves was converted into leaf discs, avoiding the main leaf vein. The leaf disc was created with a sterile punch (11 mm diameter). The leaf discs from the second group (I group) and the fourth group (SAI group) were placed in a bacterial solution (Psa, isolated and identified by our laboratory, which is listed under GenBank sequence entry number MW404385, 1–2 × 10^7^ CFU/mL) and permeated in a vacuum (until the leaf disc was completely immersed in the bacterial solution and there were no bubbles). The first group (control group) and the third group (SA group) were placed in LB liquid medium and permeated in a vacuum. Then, all leaf discs were washed three times using sterile water to remove the mucus and placed on sterilized filter paper to absorb the excess water. Finally, they were placed on a 0.8% water agar plate and in an artificial climate box (photoperiod L/D:16/8 h, diurnal temperature 16 °C/10 °C, relative humidity 95%). After treatment, samples were taken at 0 h, 24 h and 48 h, respectively, and put into sterile sample tubes. The tubes were marked, immediately frozen in liquid nitrogen and stored in the refrigerator at −80 °C, ready for use. This process was repeated 6 times for each sample.

### 4.2. Protein Extraction and Digestion

Sample analysis and protein extraction were performed using SDT (4%SDS, 100 mM Tris-HCl, 1 mM DTT, pH 7.6) buffer and quantified with the BCA Protein Assay Kit (Bio-Rad, Hercules, CA, USA). Then, the protein was digested by trypsin and desalted on C18 cartridges (Empore™ SPE Cartridges C18 (standard density), bed I.D. 7 mm, volume 3 mL, Sigma, Burlington, MA, USA), concentrated via vacuum centrifugation and reconstituted in 40 µL of 0.1% (*v*/*v*) formic acid. Filter-aided sample preparation (FASP digestion) procedure: 200 μg of protein for each sample was incorporated into 30 μL SDT buffer (4% SDS, 100 mM DTT, 150 mM Tris-HCl pH 8.0). The detergent, DTT and other low-molecular-weight components were removed using UA buffer (8 M urea, 150 mM Tris-HCl pH 8.0) via repeated ultrafiltration (Microcon units, 10 kD). Then, 100 μL iodoacetamide (100 mM IAA in UA buffer) was added to block reduced cysteine residues and the samples were incubated for 30 min in darkness. The filters were washed with 100 μL UA buffer three times and then 100 μL 25 mM NH_4_HCO_3_ buffer twice. Finally, the protein suspensions were digested with 4 μg trypsin (Promega) in 40 μL 25 mM NH_4_HCO_3_ buffer overnight at 37 °C, and the resulting peptides were collected as a filtrate. The peptides of each sample were desalted on C18 cartridges (Empore™ SPE Cartridges C18 (standard density), bed I.D. 7 mm, volume 3 mL, Sigma, Burlington, MA, USA), concentrated via vacuum centrifugation and reconstituted in 40 µL of 0.1% (*v*/*v*) formic acid. The peptide content was estimated based on the UV light spectral density at 280 nm using an extinction coefficient of 1.1 of 0.1% (g/L) solution that was calculated based on the frequency of tryptophan and tyrosine in vertebrate proteins.

### 4.3. LC-MS/MS Analysis

LC-MS/MS analysis was performed on a timsTOF Pro mass spectrometer (Bruker timsTOF Pro2, Billerica, MA, USA) coupled to Nanoelute (Bruker, Billerica, MA, USA) for 60/120/240 min. The peptides were loaded onto a reverse-phase trap column (Thermo Scientific Acclaim PepMap100, 100 μm × 2 cm, nanoViper C18, Waltham, MA, USA) connected to the C18 reverse-phase analytical column (Thermo Scientific Easy Column, 10 cm long, 75 μm inner diameter, 3 μm resin, Waltham, MA, USA) in buffer A (0.1% formic acid) and separated with a linear gradient of buffer B (84% acetonitrile and 0.1% formic acid) at a flow rate of 300 nl/min, controlled by IntelliFlow technology. The mass spectrometer was operated in positive ion mode. The mass spectrometer collected ion mobility MS spectra over a mass range of *m*/*z* 100–1700 and 1/k0 of 0.6 to 1.6 and then performed 10 cycles of PASEF MS/MS with a target intensity of 1.5 k and a threshold of 2500. Active exclusion was enabled with a release time of 0.4 min.

### 4.4. Identification and Quantitation of Proteins

The MS raw data for each sample were combined and searched using the MaxQuant 1.5.3.17 software for identification and quantitation analysis. Related parameters and instructions are as follows: the max missed cleavages was 2; the main search was 6 ppm; the first search was 20 ppm; the MS/MS tolerance was 20 ppm; the Uniprot database was used for protein identification.

### 4.5. Bioinformatic Analysis

CELLO v2.5 (http://cello.life.nctu.edu.tw/ accessed on 31 July 2022), a multi-class SVM classification system, was used to predict protein subcellular localization. The protein sequences of the selected differentially expressed proteins were locally searched using the NCBI BLAST+ client software (ncbi-blast-2.2.28+-win32.exe) and InterProScan-5.25-64.0 to find homologue sequences; then, gene ontology (GO) terms were mapped and sequences were annotated using the software program Blast2GO 6.0. The GO annotation results were plotted by R scripts. The BLAST2GO 6.0 software was used to perform functional annotation following Ye et al. [56] (2006). The DEGs of each comparison were mapped to the Kyoto Encyclopedia of Genes and Genomes (KEGG) database (http://www.genome.jp/kegg/ accessed on 31 July 2022) [57]. GO and KEGG pathway enrichment analyses were performed by clusterprofiler 2.0. Only functional categories and pathways with *p*-values under a threshold of 0.05 were considered significant.

## Data Availability

The data presented in this study are available on request from the corresponding author.

## Supplementary Materials

The following supporting information can be downloaded at: https://www.mdpi.com/article/10.3390/ijms242417448/s1.
